# Hypersensitivity of mouse NEIL1-knockdown cells to hydrogen peroxide during S phase

**DOI:** 10.1093/jrr/rru021

**Published:** 2014-04-04

**Authors:** Ryohei Yamamoto, Yukari Ohshiro, Tatsuhiko Shimotani, Mizuki Yamamoto, Satoshi Matsuyama, Hiroshi Ide, Kihei Kubo

**Affiliations:** 1 Department of Advanced Pathobiology, Graduate School of Life & Environmental Sciences, Osaka Prefecture University, 1-58 Rinku Ourai Kita, Izumisano, Osaka 598-8531, Japan; 2 Department of Mathematical and Life Sciences, Graduate School of Science, Hiroshima University, Kagamiyama, Higashi-Hiroshima, Hiroshima 739-8526, Japan

**Keywords:** BER, cell cycle, glycosylase, mouse, NEIL1

## Abstract

Oxidative base damage occurs spontaneously due to reactive oxygen species generated as byproducts of respiration and other pathological processes in mammalian cells. Many oxidized bases are mutagenic and/or toxic, and most are repaired through the base excision repair pathway. Human endonuclease VIII-like protein 1 (hNEIL1) is thought to play an important role during the S phase of the cell cycle by removing oxidized bases in DNA replication fork-like (bubble) structures, and the protein level of hNEIL1 is increased in S phase. Compared with hNEIL1, there is relatively little information on the properties of the mouse ortholog mNEIL1. Since mouse cell nuclei lack endonuclease III-like protein (NTH) activity, in contrast to human cell nuclei, mNEIL1 is a major DNA glycosylase for repair of oxidized pyrimidines in mouse nuclei. In this study, we made mNEIL1-knockdown cells using an shRNA expression vector and examined the cell cycle-related variation in hydrogen peroxide (H_2_O_2_) sensitivity. Hypersensitivity to H_2_O_2_ caused by mNEIL1 knockdown was more significant in S phase than in G1 phase, suggesting that mNEIL1 has an important role during S phase, similarly to hNEIL1.

## INTRODUCTION

Oxidized DNA damage occurs continuously in living cells due to reactive oxygen species (ROS) induced by cellular respiration or derived from extracellular sources. Such damage is mainly repaired by base excision repair (BER) initiated by excision of oxidized bases by specific glycosylases [1]. Five mammalian glycosylases are categorized into Nth and Nei families according to their structural features, and these proteins share a variety of oxidized bases as their substrates. Endonuclease VIII-like protein 1 (NEIL1) has an apurinic/apyrimidinic (AP)-lyase that cleaves 3′ and 5′ phosphodiester bonds at AP sites [2]. Human NEIL1 (hNEIL1) has a well-characterized broad range of substrates including formamidopyrimidines (Fapys) and other oxidized pyrimidines. hNEIL1 can excise base damage in duplex DNA, as well as in bubble structures [3] and single-stranded DNA [4], and thus is likely to have important roles in repair during transcription or replication. In agreement with this idea, the expression and activity of hNEIL1 are elevated in S phase, and hNEIL1 interacts with replication-related proteins such as PCNA, RPA, FEN1 and WRN helicase [5–8]. Recently, Hegde *et al.* (2013) presented a detailed model in which hNEIL1 was involved in the replication complex and had a role in prereplicative repair of oxidized bases and a proposed regulatory role in avoidance of double-strand breaks [9].

Mouse NEIL1 (mNEIL1) was discovered at about the same time as the human homolog [10], and knockout mice have been established. Studies using these mice have suggested that mNEIL1 has important roles in prevention of diseases associated with metabolic syndrome [11] and in protection of neurons against ischemic injury [12]. However, compared with hNEIL1, information on the role of mNEIL1 in DNA repair is relatively limited [10, 13–18]. In mouse cell nuclei, glycosylases for repair of oxidized DNA damage differ somewhat from those in human cell nuclei. Human endonuclease III-like protein 1 (hNTH1), a structural homolog of *Escherichia coli* endonuclease III that repairs a variety of oxidized pyrimidines including thymine glycol, is localized in nuclei, whereas mouse NTH1 (mNTH1) is predominantly localized in mitochondria [19]. Therefore, mNEIL1 and a monofunctional thymine glycol glycosylase [20] seem to be the major glycosylases for repair of oxidized pyrimidines in mouse cell nuclei. mNEIL1-depleted mouse ES cells have elevated radiosensitivity [21], and mNEIL1 knockout mouse embryonic fibroblasts (MEFs) showed hypersensitivity to hydrogen peroxide (H_2_O_2_) [22], whereas the sensitivity of germinal center B cells to H_2_O_2_ was not affected by mNEIL knockout [23]. Since hNEIL1-knockdown HEK293 cells show increased sensitivity to glucose oxidase, which generates H_2_O_2_ [24], it is important to test other types of NEIL1-knockdown mouse cells for their H_2_O_2_ sensitivity. In addition, there is no direct evidence that depletion of mNEIL1 or hNEIL1 affects the sensitivity of S-phase cells to oxidative stress, but a requirement for hNEIL1 has been shown in DNA repair during DNA replication. In the present study, we made three mNEIL1-knockdown clone cells and examined their cell cycle-dependent sensitivities to H_2_O_2_.

## MATERIALS AND METHODS

### Cell lines

Mouse embryonic fibroblasts (MEFs) and mouse L cells were generous gifts from Dr Masahiko Miura (Tokyo Medical and Dental University) and Dr Osamu Inanami (Hokkaido University), respectively. Both cell lines were cultured in Eagle's MEM ‘Nissui’ 1 (Nissui, Tokyo, Japan) supplemented with 10% fetal bovine serum (Thermo Scientific, Waltham, MA), MEM non-essential amino acids solution (Gibco BRL, Carlsbad, CA) and sodium pyruvate solution (Gibco BRL) at 37°C in 5% CO_2_.

### mNEIL1 knockdown

Knockdown target sequences were selected by siRNA Wizard software (InvivoGen, San Diego, CA) based on the mNEIL1 nucleotide sequence (NCBI: NM_028347). These sequences were located in the H2TH domain of mNEIL1. Two short hairpin oligonucleotides (Table 1) including each knockdown sequence (Sigma Aldrich, St Louis, MO) were inserted into a psiRNA-hH1GFPzeoG2 shRNA expression vector (InvivoGen). The plasmid was transfected into *Escherichia coli* JM109 by Cell-Porator^TM^ (Gibco BRL), amplified in LB medium containing 25 μg/ml Zeocin (InvivoGen), and purified using a QIAprep spin Miniprep Kit (Qiagen, Hilden, Germany). The nucleotide sequences were confirmed by EQ8000 (Beckman Coulter, Brea, CA). The plasmid was introduced into MEFs or mouse L cells using HilyMax (Dojindo, Kumamoto, Japan). Medium containing Zeocin (500 μg/ml for MEFs, 200 μg/ml for mouse L cells) was renewed every 3 or 4 d.

**Table 1.** Oligonucleotides inserted into a shRNA expression plasmid

MEF (F)	5′-ACCTC**GATCCTGTACCGGCTGAAGAT**TCAAGAGATCTTCAGCCGGTACAGGATCTT-3′
MEF (R)	5′-CAAAAAGATCCTGTACCGGCTGAAGATCTCTTGA**ATCTTCAGCCGGTACAGGATC**G-3′
L cell (F)	5′-ACCTC**GAAGGCTCGTACAGTTCTAGA**TCAAGAGTCTAGAACTGTACGAGCCTTCTT-3′
L cell (R)	5′-CAAAAAGAAGGCTCGTACAGTTCTAGACTCTTGA**TCTAGAACTGTACGAGCCTTC**G-3′

Two oligonucleotide pairs are listed. Oligonucleotides MEF (R) and L cell (R) are complementary to MEF (F) and L cell (F), respectively. Knockdown target sequences are underlined.

### Western blot analysis

Exponentially growing cells were harvested, washed in cold PBS(-), and lysed in SDS gel-loading buffer (125 mM Tris-HCl, pH 6.8, 10% 2-mercaptoethanol, 4% SDS, 10% sucrose). After electrophoresis on a 12% SDS-polyacrylamide gel, proteins were transferred onto Immobilon^TM^ Transfer Membranes (Millipore, Billerica, MA). After blocking with 5% nonfat milk in TPBS (0.1% Tween 20 in PBS(-)), the membranes were incubated with rabbit polyclonal anti-mouse NEIL1 antiserum developed against full length mouse NEIL1 with a C-terminal histidine tag (Evebioscience, Wakayama, Japan) for MEF and mouse L cell extracts. To normalize the amount of mNEIL1, monoclonal anti α-tubulin antibody (MS-581-P0, Thermo Scientific) was used to quantify the α-tubulin content. After washing with TPBS, the membrane was incubated with HRP-conjugated secondary antibody (#474-1506, KPL, Gaithersburg, MD or #172-1011, BioRad, Hercules, CA) and a chromogenic reagent (20 mM sodium phosphate, pH 6.4, 0.2 mg/ml 3,3′-diaminobenzidine, tetrahydrochloride, 0.03% H_2_O_2_). Band intensity was quantified using ImageJ software (NIH, Bethesda, MD).

### H_2_O_2_ sensitivity

H_2_O_2_ sensitivities were examined by MTS assay or colony formation assay. For MTS assay, a cell suspension (100 μl) containing 20 000 cells in Eagle's MEM without fetal bovine serum and sodium pyruvate were seeded into each well of a 96-well microplate. After incubation for 3 h, H_2_O_2_ (Wako, Osaka, Japan) was added to each well. In experiments with synchronized cells, H_2_O_2_ was added immediately after cell seeding. After 2 h at 37°C, cell survival was determined by colorimetric analysis with [3-(4,5-dimethylthiazol-2-yl)-5-(3-carboxymethoxyphenyl)-2-(4-sulfophenyl)-2H-tetrazolium, inner salt (MTS)] using CellTiter 96^®^ Aqueous One Solution (Promega, Madison, WI). For colony formation assay, 200 cells from exponentially growing culture were seeded into 60-mm dishes. After incubation for 3 h, H_2_O_2_ was added to each dish and treated for 30 min at 37°C. After incubation for 7 d, colonies were fixed with methanol, stained with 2% Giemsa solution and counted. Colony numbers were normalized to that of mock controls.

### X-ray sensitivity

Four hundred log-phase cells in Eagle's MEM were seeded into a 100-mm dish. After incubation for 3 h, cells were exposed to X-rays at 0.5, 1, 2, 4 and 6 Gy (250 kV, 14 mA, filtered with 0.3 mm Cu and 0.5 mm Al) at a dose rate of 0.465 Gy/min. After incubation for 7 d, colonies were fixed with methanol, stained with 2% Giemsa solution, and counted under a microscope. Colony numbers were normalized to that of mock-irradiated controls.

### Cell cycle synchronization by serum starvation and flow cytometry

Cells were incubated in Eagle's MEM without serum for 24 h and then in Eagle's MEM for 15 h. Mitotic cells were collected by mitotic shake off, and the cell suspension was centrifuged at 1000×g for 5 min. After washing with PBS(-), the cells were resuspended in Eagle's MEM and incubated for 3 or 10 h to obtain a G1 or S phase-dominant population, respectively. The synchronized cell suspension (1 × 10^6^ cells/ml) in PBS(-) was mixed with a 1/5 volume of 1.2% TritonX-100-containing PBS(-). After centrifugation at 3000×g for 5 min at 4°C, the nuclei precipitate was washed with PBS(-) containing 0.2% TritonX-100 and incubated in the presence of RNaseA (50 μg/ml) at 37°C for 30 min. After centrifugation at 3000×g for 5 min at 4°C, the nuclei pellet was washed with PBS(-) containing 0.2% TritonX-100 and resuspended and stained in PBS(-) containing 0.2% TritonX-100 and 10 μg/ml PI at 37°C for 20 min. After passing through nylon mesh, nuclei were analyzed using a FACS Calibur (Becton-Dickinson, Franklin Lakes, NJ).

## RESULTS

### mNEIL1-knockdown cells show hypersensitivity to H_2_O_2_

**Fig. 1.**
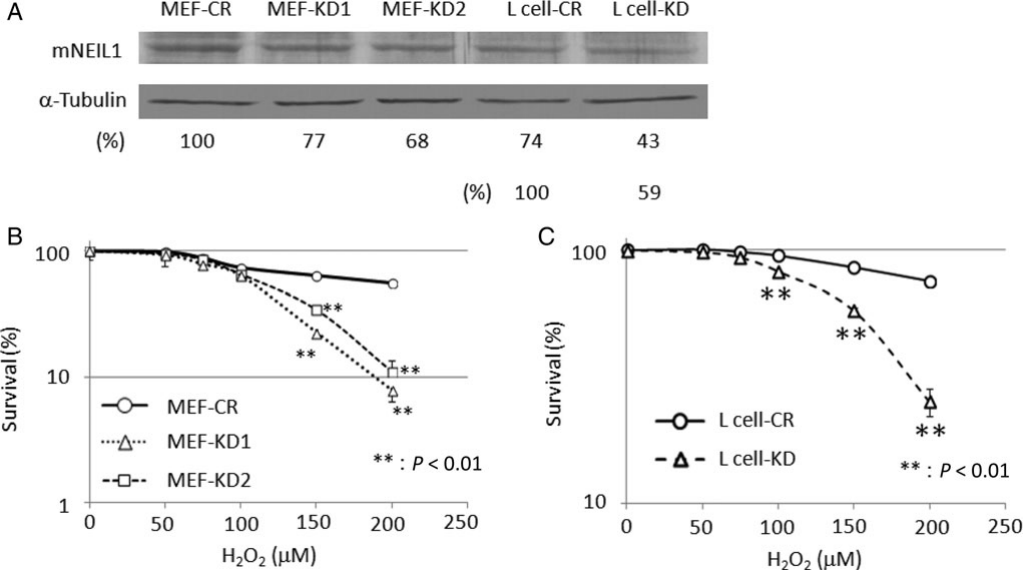
Cellular mNEIL1 protein levels and H_2_O_2_ sensitivities of mNEIL1-knockdown MEFs and mouse L cells. (**A**) Western blotting analyses of mNEIL1 protein expression. The relative mNEIL1 levels in the knockdown clones are shown. The mNEIL1 expression levels in knockdown MEFs (MEF-KD1, MEF-KD2) and mouse L cells (L cell-KD) were 77, 68 and 59%, respectively, compared with the respective control cells (MEF-CR and L cell-CR). (**B**, **C**) Survival rates of MEFs (B) and mouse L cells (C) after H_2_O_2_ treatment were determined by MTS assay. Details are described in Materials and Methods. Each point is the average of four experiments, and error bars represent the standard deviation from the mean. Error bars are shown when larger than symbols. Asterisks represent statistically significant difference (*P* < 0.01). Knockdown of mNEIL1 increased the sensitivities of both strains to H_2_O_2_.

mNEIL1-knockdown cells were prepared by lipofection of psiRNA-hH1GFPzeoG2 shRNA expression vector into MEFs or mouse L cells. After cloning each knockdown cell, western blotting analyses of whole cell extracts from the cloned cells were performed. The expression levels of mNEIL1 protein were 77 and 68% in two knockdown MEFs (MEF-KD1 and MEF-KD2, respectively) and 59% in mouse L cells (L cell-KD), compared with control cells with empty vectors (Fig. 1A). The knockdown cells showed no change in morphology under a microscope and no change in proliferation potential. An MTS assay was performed to investigate sensitivity to an oxidizing agent. Cells were treated with H_2_O_2_ at 37°C for 2 h in Eagle's MEM without FBS and sodium pyruvate. The two knockdown MEFs and the knockdown mouse L cells showed hypersensitivity at high H_2_O_2_ concentration, compared with control cells (Fig. 1B and C).

### Knockdown cells expressing two-thirds of mNEIL1 show no hypersensitivity to X-rays

**Fig. 2.**
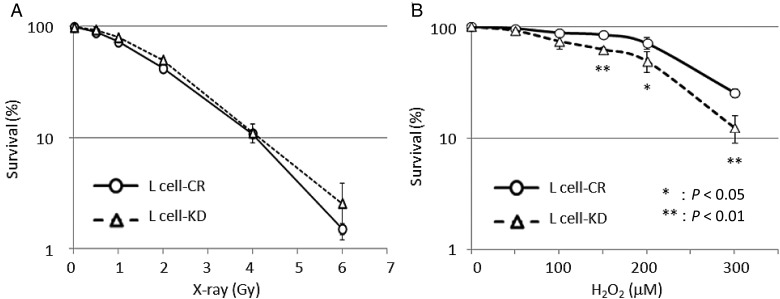
X-ray and H_2_O_2_ sensitivities of mNEIL1-knockdown mouse L cells measured by colony formation assay. Colony numbers after X-ray irradiation (**A**) or H_2_O_2_ treatment (**B**) were represented as a percentage of the value at mock treatment. Each point is the average of three experiments, and error bars represent the standard deviation from the mean. Error bars are shown when larger than symbols. Knockdown of mNEIL1 increased the sensitivity to H_2_O_2_ but showed no significant effect on X-ray sensitivity.

Rosenquist *et al.* reported that the mouse cell lines showing a 5-fold reduction in mNEIL1 relative to wild-type were hypersensitive to gamma-rays [21]. However, there was no significant difference in colony-forming abilities after X-irradiation (0∼6 Gy) between control and knockdown cells (Fig. 2A), while a significant difference was observed in H_2_O_2_-treated cells (Fig. 2B).

### H_2_O_2_ hypersensitivity of mNEIL1-knockdown cells is more significant in S phase than in G1 phase

**Fig. 3.**
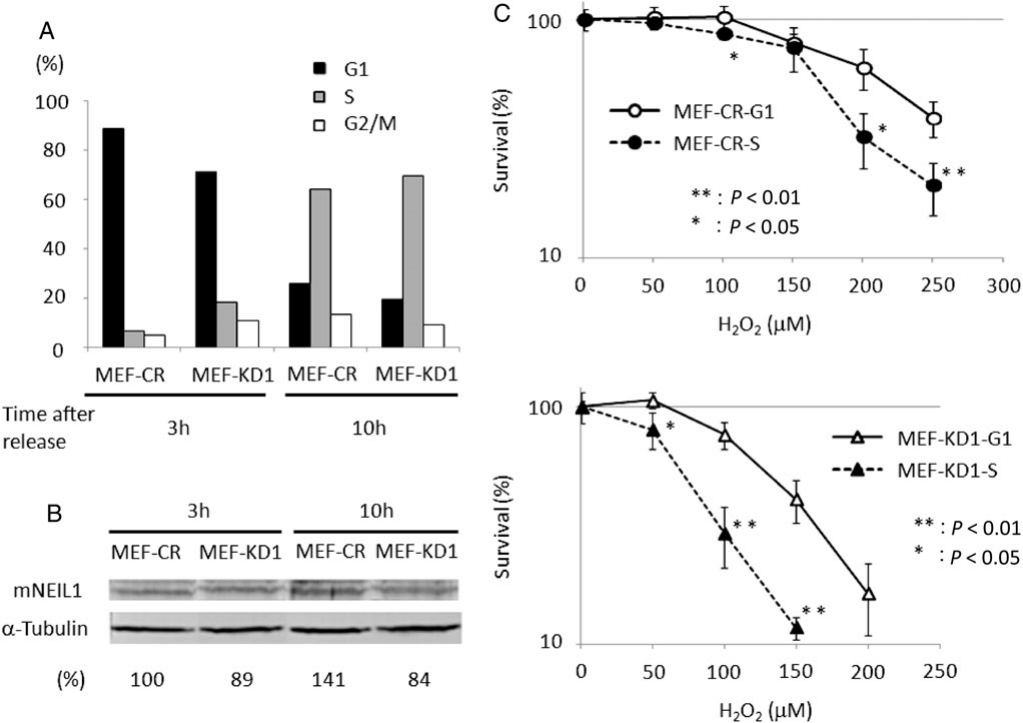
H_2_O_2_ sensitivity of MEF at G1 or S phase. (**A**) Synchronization of MEF was performed by serum starvation. Black, gray and white columns represent cell proportions at G1, S and G2/M phase, respectively. At 3 and 10 h after release from the growth arrest, ∼80% and ∼70% of cells were in G1 and S phase, respectively. (**B**) Western blot analysis of mNEIL1 levels in G1 and S phase cells. (**C**) Survival ratios of MEF at each cell cycle stage after H_2_O_2_ treatment were determined by MTS assay. Each point is the average of four experiments, and error bars represent the standard deviation from the mean. Both MEF-Control and MEF-Knockdown at S phase were more sensitive than at G1 phase. Furthermore, the difference in survival was more significant in MEF-Knockdown than MEF-Control.

It is likely that hNEIL1 has an important role during S phase, but the effect of hNEIL1 depletion on sensitivity to oxidative stress in S phase has not been investigated directly. Therefore, the hypersensitivity to H_2_O_2_ of mNEIL1-knockdown MEFs was examined in S and G1 phases. At 3 h after release from serum starvation ∼80% of the cells were in G1 phase, and at 10 h ∼70% were in S phase (Fig. 3A). The mNEIL1-expression level of the cell population at each phase was calculated by western blot analysis. While the mNEIL1 expression level in control cells in S phase was 1.4-fold higher than that in G1 phase (Fig. 3B), no such difference was observed in knockdown cells. Both knockdown and control cells in S phase were more hypersensitive to H_2_O_2_ compared with those in G1 phase (Fig. 3C). However, the difference in survival of H_2_O_2_-treated G1 and S phase cells was only apparent at over 200 μM H_2_O_2_ in control cells, but was significant at as low as 50 μM H_2_O_2_ in knockdown cells. These results suggest that mNEIL1 plays a more important role in S phase than in G1 phase.

## DISCUSSION

Murine NEIL1 is a bifunctional DNA glycosylase that acts on formamidopyrimidines and oxidized pyrimidines, with accompanying AP lyase activity that cleaves 3′ and 5′ phosphodiester bonds at AP sites to leave a single nucleotide gap and initiate the APE1-independent BER pathway. hNEIL1 has well-characterized interactions with proteins involved in cell cycle regulation, DNA replication and BER. Since these interactions take place with the common C-terminal domain of hNEIL1, it is conceivable that there is an underlying mechanism of regulation of hNEIL1 activity. Of the cell cycle checkpoint proteins, the Rad9-Rad1-Hus1 (9-1-1) heterotrimer and each monomer protein of 9-1-1 stimulate hNEIL1 activity and interact with the hNEIL1 C-terminal domain [25]. With regard to DNA replication, RPA downregulates hNEIL1 activity for single-strand (SS) substrates and activates hNEIL1 activity for duplex substrates [6].

Several BER proteins, including DNA polymerase β, XRCC1, DNA ligase IIIα and FEN-1, have also been shown to interact with hNEIL1 [5–8, 24, 26], with the C-terminal domain of hNEIL1 serving as a common interaction domain. Interaction of hNEIL1 with poly (ADP-ribose) polymerase-1 (PARP-1) [27], a DNA damage sensor, has been observed *in vitro* and *in vivo*, and interaction of the C-terminal region of hNEIL1 and the BRCT domain of PARP1 inhibits hNEIL1 incision activity in a concentration-dependent manner. These results suggest that hNEIL1 is not a simple glycosylase/AP-lyase, but plays multiple roles in a highly organized DNA damage control system.

In this study, both mNEIL1-knockdown MEFs and L cells showed significantly increased sensitivity to H_2_O_2_ (Fig. 1), whereas the radiosensitivity of these cells did not change (Fig. 2). It is possible that the difference in hypersensitivity between X-rays and H_2_O_2_ might result from the type of DNA damage formed and/or the knockdown level being insufficient to affect the radiosensitivity. It has been reported that mNEIL1 knockdown cell lines occasionally recover their original radiosensitivity after prolonged cultivation [21]. However, our knockdown cells were stable enough to show hypersensitivity to H_2_O_2_ over 12 passages. In the synchronized population, the effect of mNEIL1 knockdown on H_2_O_2_ sensitivity was more significant in S phase cells than in G1 cells (Fig. 3). This result clearly indicates that mNEIL1 plays a pivotal role in defense against oxidative damage in S phase. This finding seems to be consistent with experimental data for hNEIL1, suggesting that mNEIL1 has similar roles to those of hNEIL1. Surprisingly, although MEF-KD-G1 cells showed only 11% reduction of mNEIL1 compared with MEF-CR-G1, the MEF-KD-G1 cells showed higher sensitivity to H_2_O_2_ than the MEF-CR-G1 cells. It seems to be that the increased sensitivity resulted from a significant number of S phase cells in the MEF-KD1-G1 cell population. Furthermore, although the mNEIL1 expression level in MEF-KD cells was higher than that in L cell-KD cells, as shown in Fig. 1, the survival rate of MEF-KD cells was lower than that of L cell-KD cells, suggesting that mNEIL1 reduction may be very effective in MEF. As mentioned above, hNTH1 shares the responsibility for repair of oxidized pyrimidines with hNEIL1 in human cell nuclei. Since mNTH1 is mainly localized in mitochondria, a monofunctional thymine glycol glycosylase may take its place in mouse cell nuclei [20]. This monofunctional glycosylase can cover the absence of mNTH1 in nuclei, but we conclude that reduction of the mNEIL1 level to two-thirds of that in wild-type cells has a significant effect on cellular defense against oxidative threats, presumably because of the different substrate specificity of mNEIL1 and its regulatory role in DNA repair.

## FUNDING

This work was supported in part by the Japan Society for the Promotion of Science (JSPS) Grant-in Aid for Scientific Research (KAKENHI) Grants No. 20510054 and No. 25340036.
